# Identification and Functional Characterization of a Novel Insecticidal Decapeptide from the Myrmicine Ant *Manica rubida*

**DOI:** 10.3390/toxins11100562

**Published:** 2019-09-25

**Authors:** John Heep, Marisa Skaljac, Jens Grotmann, Tobias Kessel, Maximilian Seip, Henrike Schmidtberg, Andreas Vilcinskas

**Affiliations:** 1 Branch for Bioresources, Fraunhofer Institute for Molecular Biology and Applied Ecology (IME), Winchesterstrasse 2, 35394 Giessen, Germany; John.Heep@ime.fraunhofer.de (J.H.); Marisa.Skaljac@ime.fraunhofer.de (M.S.); Jens.Grotmann@ime.fraunhofer.de (J.G.); Tobias.Kessel@ime.fraunhofer.de (T.K.); Maximilian.Seip@ime.fraunhofer.de (M.S.); 2 Institute for Insect Biotechnology, Justus Liebig University of Giessen, Heinrich-Buff- Ring 26-32, 35392 Giessen, Germany; Henrike.Schmidtberg@agrar.uni-giessen.de

**Keywords:** mass spectrometry, LC-MS, Formicidae, Myrmicinae, *Myrmica rubra*, venom gland, bioinsecticide, antimicrobial peptide, aphids, *Acyrthosiphon pisum*

## Abstract

Ant venoms contain many small, linear peptides, an untapped source of bioactive peptide toxins. The control of agricultural insect pests currently depends primarily on chemical insecticides, but their intensive use damages the environment and human health, and encourages the emergence of resistant pest populations. This has promoted interest in animal venoms as a source of alternative, environmentally-friendly bio-insecticides. We tested the crude venom of the predatory ant, *Manica rubida*, and observed severe fitness costs in the parthenogenetic pea aphid (*Acyrthosiphon pisum*), a common agricultural pest. Therefore, we explored the *M. rubida* venom peptidome and identified a novel decapeptide U-MYRTX-MANr1 (NH_2_-IDPKVLESLV-CONH_2_) using a combination of Edman degradation and de novo peptide sequencing. Although this myrmicitoxin was inactive against bacteria and fungi, it reduced aphid survival and reproduction. Furthermore, both crude venom and U-MYRTX-MANr1 reversibly paralyzed injected aphids and induced a loss of body fluids. Components of *M. rubida* venom may act on various biological targets including ion channels and hemolymph coagulation proteins, as previously shown for other ant venom toxins. The remarkable insecticidal activity of *M. rubida* venom suggests it may be a promising source of additional bio-insecticide leads.

## 1. Introduction

Ants (Hymenoptera: Formicidae) are a taxonomically diverse group of insects with more than 13,500 extant species [1]. They have evolved a venom apparatus derived from the ancestral reproductive system [2], but in contrast to other venomous phyla (e.g., snakes, spiders and scorpions) there have been few studies of ant venoms to functionally characterize their components [3]. This reflects the challenging taxonomy of ants, the limited amount of venom that can be extracted, and the common misconception that ant venoms are simple, consisting mainly of formic acid [4,5].

Recent transcriptomic and proteomic studies have shown that ant venoms are mixtures of many bioactive molecules with an impressive array of biological properties [2]. Although rich in short linear peptides (<5 kDa), ant venoms also contain complex peptides with disulfide bonds, as well as oligomeric proteins with a broad spectrum of activities [2,6,7,8,9,10]. Examples of linear peptides include dinoponeratoxins from the giant neotropical hunting ant, *Dinoponera australis* [11], ponericins from the predatory ant, *Neoponera goeldii* [12,13], and bicarinalin from *Tetramorium bicarinatum* [7]. These are classed as antimicrobial peptides (AMPs) because they show activity against microbial pathogens, but they may also possess paralytic, cytolytic, hemolytic and/or insecticidal properties [6,7,12,14,15], as ponericins do [12]. 

Insect pests reduce crop yields by feeding and transmitting plant pathogens, but they also act as vectors for many human and livestock pathogens [16,17]. Despite the increasing use of biological control methods, chemical insecticides remain the primary strategy for pest control in both agricultural and public health settings [18,19]. Over the past decade, the number of registered chemical insecticides has fallen due to the emergence of resistant pest populations and the de-registration of key insecticides by regulatory authorities, based on evidence of harm to the environment, to beneficial organisms, and to human health [20,21,22,23]. This has increased the demand for new and safe insecticidal lead compounds, novel insecticidal targets, and alternative methods for effective pest control. 

Venom-derived peptidyl toxins have been fine-tuned by evolution to improve their selectivity and efficacy in the context of prey capture and defense against predators [2]. Insecticidal toxins derived from predatory arthropods therefore offer a promising source of novel bio-insecticides, and these frequently include peptidyl neurotoxins derived from venoms of arachnids, such as scorpions and spiders [24,25]. Ants also use venom for predation and/or defense against predators and competitors. For example, the linear peptide, poneratoxin, isolated from *Paraponera clavata*, targets the central nervous system of insects by blocking synaptic transmission [2,26,27]. In contrast, peptides with disulfide bonds often target ion channels, causing paralysis or incapacitation. For example, poneritoxin Ae1a from the predatory ant, *Anochetus emarginatus*, and MIITX_1_-Mg1a from the giant red bull ant, *Myrmecia gulosa*, antagonize ion channels in sheep blowflies (*Lucilia cuprina*) and crickets (*Acheta domesticus*) [4,28].

Aphids (Hemiptera: Aphididae) are devastating insect pests that damage plants by feeding and by transmitting many important plant pathogens [19,29,30,31]. Aphid control relies predominantly on chemical insecticides such as carbamates, organophosphates, neonicotinoids and pyrethroids [18,29,32]. These insecticides mainly act on insect nerve and muscle targets, whereas other pesticides impair respiration, lipid synthesis, or cuticle formation during insect growth and development [18]. The long-term and frequent use of these insecticides have applied selective pressure to aphid populations, and multiple resistant forms have emerged, making some aphid species very difficult to control [18,33]. The polyphagous aphids, *Myzus persicae* and *Aphis gossypii*, are among the 12 most resistant insect species, with resistance to 75 and 48 chemical insecticidal compounds, respectively [18]. 

Recent studies have shown that both linear and disulfide-bonded toxins from venoms of spiders, scorpions, and ants are promising molecules for aphid control [34,35,36,37,38,39]. Some peptidyl toxins from scorpions (e.g., *Urodacus yaschenkoi* and *Urodacus manicatus*) and the ant *Myrmica rubra* act against aphids as stand-alone compounds [34,39]. Other scorpion and spider toxins are more active when fused to carrier proteins (e.g., *Galanthus nivalis* agglutinin) that mediate transport through the insect gut [37] or by engineering entomopathogenic fungi to express insecticidal proteins [36]. 

*Manica rubida* (Myrmicinae) is a stinging ant of moderate size (workers ~6–9 mm in length) that is prevalent in middle and southern Europe [40], favoring mountainous regions of 500–2000 m altitude [41]. Colony founding in *M. rubida* is semi-claustral [42], a strategy in which the founding queen actively preys upon other insects to enhance early colony development [42]. This species inflicts a painful sting and uses its potent venom to prevent nest invasions and to subdue prey [40]. Although *M. rubida* venom contains predominantly small, linear peptides (<5 kDa) [43], the peptide sequences are unknown and their activities and that of the crude venom have not been investigated in detail.

We explored the *M. rubida* venom peptidome to identify new peptides, using a combination of liquid chromatography/mass spectrometry (LC-MS) and Edman degradation. We also isolated one of the most abundant linear peptides in the crude venom (U-MYRTX-MANr1) and tested its antimicrobial activity and potency against the pea aphid (*Acyrthosiphon pisum*), a common agricultural pest.

## 2. Results

### 2.1. LC-MS Analysis of Crude Venom

**Figure 1:**
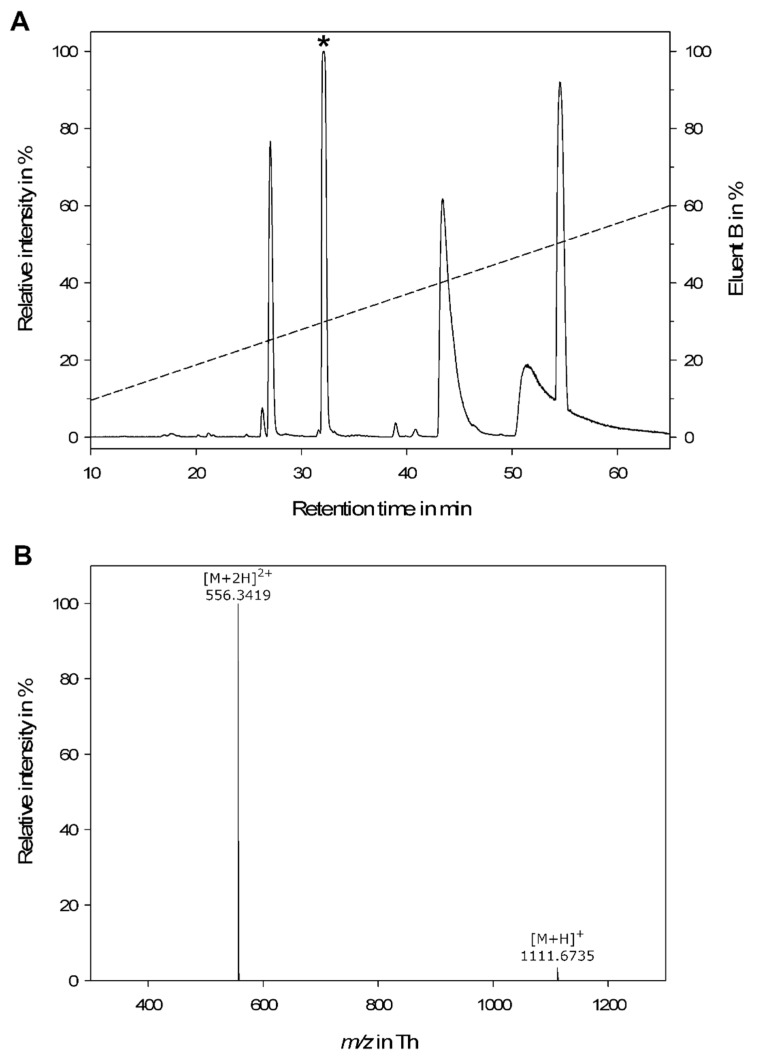
(**A**) Crude venom of the myrmicine ant, *Manica rubida*, was analyzed by LC-MS on a C_18_ reversed-phase column with gradient elution (dashed line) using water and acetonitrile supplemented with 0.1% formic acid as mobile phases. The asterisk indicates the peptide that was isolated for further characterization. (**B**) The mass spectrum of the corresponding decapeptide U-MYRTX-MANr1 acquired on a high-resolution micrOTOF-QII instrument (Bruker Daltonics, Billerica, MA, USA).

Pooled *M. rubida* worker venom was analyzed by LC-MS. We identified 180 molecular features representing a set of 96 different peptides, most of which eluted between 25 and 55 min (23–50% acetonitrile) revealing low to moderate hydrophobicity (Figure 1). The mass range of the peptides was 368–3026 Da, representing sequences of 3–27 amino acids (Figure S1). The sequence length was calculated using an average amino acid molecular mass of 111.1254 Da [44].

Crude venom was reduced and alkylated to identify peptides with disulfide bonds. Among the most abundant peptides in the venom peptidome (relative peak intensity ≥ 3%), linear peptides were prevalent, but there were also some peptides with a single, intramolecular disulfide bond (Table 1).

### 2.2. Peptide Sequencing

A fraction from the crude venom containing a peptide with a molecular weight of 1110.67 Da, eluting at 32.1 min, was isolated and enriched. This fraction was selected for further analysis because it contained no co-eluting peptides.

The amino acid sequence of the peptide was determined using a combination of Edman degradation and de novo peptide sequencing. Stepwise Edman degradation revealed the decapeptide sequence IDPKVLESLV (monoisotopic mass = 1111.65 Da). The difference of ~1 Da between the theoretical and experimental masses reflected C-terminal amidation. The structure of the novel peptide was confirmed by mass determination in the sub-3 ppm region and de novo peptide sequencing. The MS/MS fingerprint of the natural peptide and a synthetic analog achieved very high sequence coverage (Figure S2) and produced an exact match in terms of retention time.

The peptide was named U-MYRTX-MANr1, according to the proposed nomenclature system for ant venom peptides [2]. We added the tag “MAN” for *Manica* to avoid ambiguity, given the existence of several genera with similar names (e.g., *Myrmica, Malagidris* and *Mayriella*). The tag “r” is sufficient to denote the specific epithet, *rubida* within the genus *Manica*. The prefix “U” was added to indicate that the biological activity and pharmacological target of the peptide remain unidentified [45].

### 2.3. Antimicrobial Activity

Synthetic analogs of the decapeptide U-MYRTX-MANr1 (*M. rubida*) and the decapeptide U-MYRTX-MRArub1 from the myrmicine ant, *M. rubra* [39], were tested in antimicrobial assays against a range of bacteria and fungi. Neither decapeptide showed any activity against any strains we tested at concentrations up to 512 µg·mL^−1^. 

### 2.4. Effects of Crude Venom and Peptidyl Toxins on Aphid Survival and Reproduction

**Figure 2:**
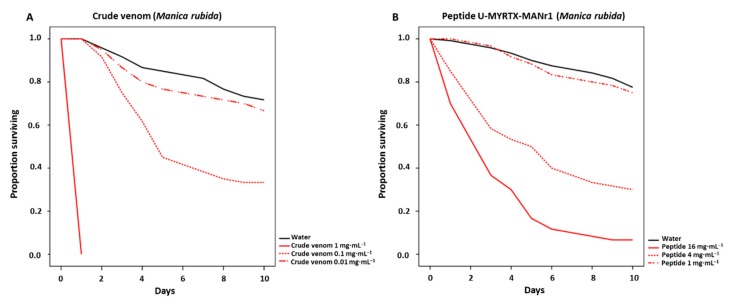
Low pea aphid survival reveals the strong insecticidal activity of crude *Manica rubida* venom (100% mortality) and peptide U-MYRTX-MANr1 at high (~93% mortality) and medium concentrations (~30% mortality) after 10 days. Survival (60 aphids in three biological replicates of 20 individuals per treatment) was monitored for 10 days following the injection of crude venom (**A**) or the peptide (**B**) into pea aphids, *Acyrthosiphon pisum*. Survival data were analyzed using Kaplan–Meier statistics and comparisons between the treatment and control were based on log-rank tests. Statistical data are shown in Table 2 and Table S2.

Insecticidal activities of crude *M. rubida* venom and the peptide U-MYRTX-MANr1 were determined by tracking *A. pisum* survival (Figure 2) and offspring production daily until 10 days post-injection (Table S1). In a previous study, the *M. rubra* peptide U-MYRTX-MYRrub1 was active against *A. pisum* [39]. Therefore, crude *M. rubra* venom and U-MYRTX-MYRrub1 were used as positive controls to determine the relative activity of *M. rubida* and *M. rubra* venom components on aphid fitness (Figure S3, Table S1).

In most treatments, aphid survival was reduced in a concentration-dependent manner by the crude venom and peptidyl toxins from both species (Table 2). *M. rubida* venom was more potent than *M. rubra* venom (Table 2, Tables S2 and S3; Figure 2 and Figure S3). The highest concentration (1 mg·mL^−1^) of *M. rubida* venom caused 100% mortality within 24 h, whereas ~20% of aphids were still alive 10 days after exposure to the same concentration of *M. rubra* venom (Figure 2A and Figure S3A). Furthermore, *M. rubida* and *M*. *rubra* venoms at a concentration of 0.1 mg·mL^−1^ reduced aphid survival to ~72% and ~30%, respectively (Table 2). All concentrations of the bovine serum albumin (BSA) control (1, 4 and 16 mg·mL^−1^) and low concentrations of crude venom (0.01 mg·mL^−1^) or peptide (1 mg·mL^−1^) from either species had no significant effect on aphid survival (Table 2 and Table S4; Figure 2, Figures S3 and S4).

To determine the effect of venom/peptide injection on aphid reproduction, the number of offspring was scored for aphids that survived the treatments. Due to the strong effect of high concentrations of the crude venom/peptide on aphid survival, we only counted the number of offspring for aphids exposed to medium and low concentrations (Table S1). Medium concentrations of crude venom (0.1 mg·mL^−1^) and peptidyl toxin (4 mg·mL^−1^) from *M. rubida* both reduced the number of offspring by ~26%, whereas other treatments, including all treatments with the *M. rubra* venom/peptide, did not reduce aphid reproduction (Table S1). 

### 2.5. Effects of Crude Venom and Peptide Toxins on Aphid Behavior and Injection Wound Healing

**Figure 3:**
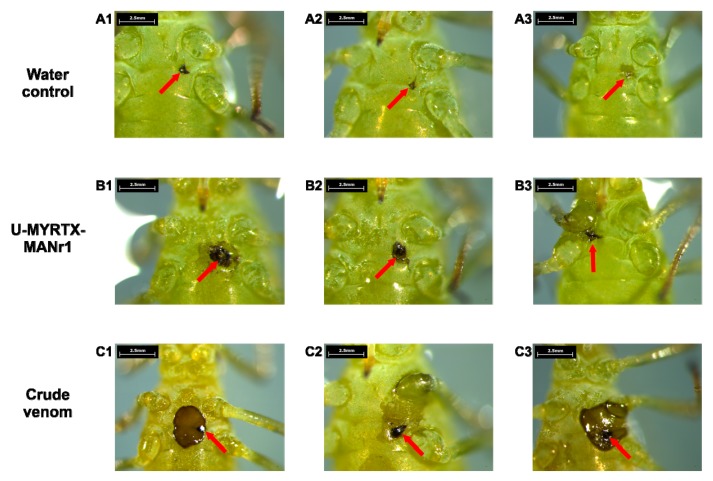
Injection wound healing in *Acyrthosiphon pisum* 1 h post-injection with water (A1–A3), U-MYRTX-MANr1 (16 mg·mL^−1^) (B1–B3), or crude *Manica rubida* venom (1 mg·mL^−1^) (C1–C3) (30 individuals injected per treatment). Hemolymph coagulation was impaired in aphids injected with U-MYRTX-MANr1, but the effects were more severe with crude venom, resulting in a massive loss of hemolymph. The injection site and melanization of exposed hemolymph are indicated with red arrows.

The strong toxicity of *M. rubida* crude venom and toxin U-MYRTX-MANr1 on aphid survival and reproduction persuaded us to investigate the effects of crude venom or toxin on the behavior of *A. pisum* immediately and 1 h after injection (Table 3). Interestingly, aphids placed on their backs immediately after injection with crude *M. rubida* venom displayed an inability or delayed ability (>3 min) to right themselves. Similar, but much less severe effects, were observed following the injection of U-MYRTX-MANr1 (Table 3). Aphids in the control group (water injection) showed normal movement and rapidly righted themselves when placed on their backs. Observations recorded for crude venom, peptide, and water immediately after injection remained unchanged after 1 h, but a massive loss of body fluids from the injection site occurred in aphids treated with *M. rubida* crude venom and U-MYRTX-MANr1 (Figure 3, B1–C3). This suggests that both the venom and the peptide impair wound healing and hemolymph coagulation. 

To understand the effectiveness of *M. rubida* venom components in more detail, injections using crude venom and toxin U-MYRTX-MYRrub1 from *M. rubra* were repeated. In contrast to injections of *M. rubida* venom, paralysis and fluid loss were significantly less severe for both crude venom and the *M. rubra* toxin (Table S5). Nevertheless, we observed differences between control aphids and those injected with crude *M. rubra* venom or peptidyl toxin (Figure S5, Table S5). Aphids injected with crude *M. rubra* venom experienced mild paralysis and required 10–30 s to right themselves. Paralytic effects were still evident 1 h post-injection, and were associated with mildly impaired wound healing/coagulation (Figure S5, C1–C3). In agreement with the *M. rubida* experiments, the effects on movement and wound healing/coagulation were weaker for toxin U-MYRTX-MYRrub1 than for crude venom. Aphids injected with the peptide were able to right themselves immediately and showed only mild paralysis (Table S5). Furthermore, the mild paralysis was maintained 1 h post-injection and wound healing/coagulation was only slightly impaired (Figure S5, B1–B3). 

To further investigate the effectiveness of *M. rubida* venom components against aphids, histological investigations were also conducted. Histological sections of aphids injected with crude *M. rubida* venom showed the significant retardation of embryonic development, degradation of fat bodies, and dissociation of bacteriocytes (Figure S6). In addition, impaired wound healing following crude *M. rubida* venom injections was confirmed, as observed in other injection assays (Figure 3, C1–C3; Figure S6). Histological sections of aphids injected with water and peptide U-MYRTX-MANr1 did not show obvious effects (Figures S6–S8). Aphids injected with *M. rubra* venom components were not subjected to histological investigation. 

### 2.6. Effects of Peptide U-MYRTX-MANr1 on Aphid Susceptibility to Chemical Insecticides

The oral activity of toxin U-MYRTX-MANr1 was determined by tracking aphid survival during 3 days of feeding (Figure S9). Aphid survival was not significantly affected following oral delivery of the peptide at a concentration of 500 µg·mL^−1^, as used in previous studies [34,39]. Aphids that survived 3 days on the control or peptide-supplemented diet were exposed to insecticide-treated bean plant leaf discs to determine the effect of U-MYRTX-MANr1 on aphid tolerance to three frequently used chemical insecticides: imidacloprid, spirotetramat, and methomyl. Each insecticide was tested at a sub-lethal concentration, determined in a previous study [46] (Table S6). Aphids previously exposed to peptide U-MYRTX-MANr1 were not significantly more sensitive to any of the chemical insecticides, compared to control aphids (Table S6). The peptide U-MYRTX-MYRrub1 from *M. rubra* was not included in this experiment because its effect on the susceptibility of *A. pisum* to chemical insecticides was previously reported [39].

## 3. Discussion

*Manica rubida* is not very aggressive, but workers respond promptly to disturbances and defend their colonies with powerful stings, which can be painful to humans. Although very little is known about the natural feeding preferences of *M. rubida*, N-isotope data have shown that this species is zoophagous [47]. This indicates that these ants are predominately scavengers and predators, using their potent venom to subdue prey. Our LC-MS analysis of *M. rubida* venom revealed significant heterogeneity of the venom peptidome, with numerous peptides varying in molecular weight, structure, and physochemical properties. We also determined the first sequence of a peptidyl toxin isolated from *M. rubida* venom and characterized its biological activity.

Ant venoms are widely assumed to be simple mixtures, consisting mainly of formic acid, but this is only true for species of the subfamily *Formicinae* [4]. In contrast, species such as *M. gulosa* [4] and *Odontomanchus monticola* [48] produce venoms comprising complex mixtures of peptides with diverse biological activities, which is also true of other subfamilies (e.g., *Myrmeciinae*, *Ponerinae* and *Myrmicinae*) [7,49,50,51]. 

Touchard et al. [43] investigated venom characteristics of *M. rubida* and 81 other stinging ant species using MALDI-TOF-MS. Our LC-MS analysis of *M. rubida* crude venom is broadly consistent with these earlier results, revealing that its venom is a heterogeneous mixture of 96 distinct peptides. Interestingly, peptide lengths and molecular weights fall within a remarkably narrow window (Figure S1), ranging from 368 to 3026 Da, with the vast majority (82 peptides, 85.4%) between 1000 and 3000 Da and 9–27 amino acids (Figure S1). The average peptide in crude *M. rubida* venom has a molecular weight of 1855 (mean) or 1965 Da (median) and comprises 17 or 18 amino acids (Figure S1). Reduction and alkylation revealed that most peptides are linear and cysteine-free (Table 1). Surprisingly, the three most abundant peptides, all of which have a single intramolecular disulfide bond, co-eluted, suggesting that they have similar amino acid compositions. These findings support our earlier observations [39] and those of others [43] concerning the venom of the ruby ant, *M. rubra*, which consists predominantly of linear peptides with molecular weights of 1000–3500 Da. Indeed, small linear peptides in the 500–4000 Da range predominate in the venoms of many ants [43]. Furthermore, peptides from cone snails (*Conus* spp.) are also small (12–30 amino acids), but in contrast to those found in ant venoms, peptides from cone snails tend to be extremely rich in disulfide bonds [52]. Peptidyl toxins from snakes or scorpions are typically longer than those of ants, varying between 40 and 100 amino acids [53]. Even so, ant peptides could nevertheless possess interesting and useful biological activities.

Although the landscape of *M. rubida* venom peptides has been investigated, no studies have attempted to characterize individual peptide toxins. We used a combination of Edman degradation, accurate mass measurement, and de novo peptide sequencing to reveal the decapeptide sequence NH_2_-IDPKVLESLV-CONH_2_, and named this peptide, U-MYRTX-MANr1, according to current nomenclature rules for ant venom peptidyl toxins [2]. This peptide features mostly (60%) hydrophobic amino acids, but also contains both acidic (aspartic acid, glutamic acid) and basic (lysine) residues, resulting in amphipathic properties. Characteristics most often associated with anti-microbial peptides (AMPs) are amphipathicity, hydrophobicity and the presence of (multiple) positive charges [54], enabling them to pass through the bacterial outer membrane, initiated by attachment to anionic lipopolysaccharides [55]. U-MYRTX-MANr1 has a net neutral charge, which may explain its lack of antimicrobial activity in vitro. This result was supported by in silico predictions of negligible antimicrobial activity when the sequence was screened against the Database for Antimicrobial Activity and Structure of Peptides [56]. The absence of antimicrobial activity was also reported for the structurally similar peptide toxins U-MYRTX-MYRrub1 from the ruby ant *M. rubra* (sequence identity 80%, sequence similarity 100%) [39] and temporin-H, a so-called AMP isolated from the defensive skin secretions of the frog *Rana temporaria* (sequence identity 40%, sequence similarity 80%) [57]. 

A full understanding of the antimicrobial potential of U-MYRTX-MANr1 will require further studies to define its activity on bacterial membranes, as well as tests against a wider panel of microbes. A multiple alignment of U-MYRTX-MANr1 with other AMPs and peptide toxins, especially ant venom peptides, is shown in Figure S10. U-MYRTX-MANr1 and U-MYRTX-MYRrub1 are from different species of the Myrmicinae, but share a highly conserved domain, with ^1^Ile ^2^Asp ^3^Pro ^4^Lys ^6^Leu ^7^Glu ^8^Ser ^9^Leu as a common motif. They differ only at positions 5 (Val→Leu) and 10 (Val→Ala), and both substitutions are conservative (hydrophobic). Interestingly, this motif seems to be unique among peptide domains discovered thus far in ant venoms, with little structural conservation compared to other ant venom peptides such as pilosulins [51] and ponericins [12]. However, the recently discovered myrmicitoxin U_12_-MYRTX-Tb1a from the myrmicine ant, *Tetramorium bicarinatum*, is an exception, in that it also shares the ^3^Pro ^6^Leu ^8^Ser ^9^Leu motif with U-MYRTX-MANr1 and U-MYRTX-MYRrub1. Such sequence alignments must be approached with some caution because only a limited number of peptide sequences from ant venom are known thus far. Further research is needed to characterize additional ant venom peptides to understand their structure–function relationships and modes of action. 

Peptide toxins isolated from scorpion and spider venoms have exhibited diverse biological activities against aphids [34,35,36,37,38]. We tested *M. rubida* crude venom and the peptide U-MYRTX-MANr1 against *A. pisum*, expanding the portfolio of peptides that may be suitable for pest management applications. Both the crude venom and the peptide U-MYRTX-MANr1 significantly reduced aphid survival and reproduction (Table 2 and Table S1; Figure 2). Interestingly, the closely related peptide U-MYRTX-MYRrub1 from *M. rubra* [39] had lower potency against aphids than U-MYRTX-MANr1 (Table 2 and Table S1, Figure S3). These peptides differ at only two residues (Figure S10), but such small differences have a profound effect on bioactivity [58]. For example, the AMPs UyCT1 and D5 derived from the scorpion *Urodacus yaschenkoi* differ at only two residues, but likewise show vastly different activities against *A. pisum* [34]. 

Our previous study showed that U-MYRTX-MYRrub1 is orally active against *A. pisum* and that sublethal concentrations of this peptide increased the susceptibility of aphids to chemical insecticides [39]. Interestingly, although injected U-MYRTX-MANr1 was more potent than U-MYRTX-MYRrub1, it did not show any direct oral effect and it did not influence the insecticide susceptibility of *A. pisum* (Figure S6, Table S6). Orally delivered scorpion AMPs isolated from *U. yaschenkoi* and *U. manicatus* show remarkable activity against aphids, but this is probably associated with their net positive charge and non-selectivity for cell membranes [58,59]. The natural properties of U-MYRTX-MANr1 suggest that its insecticidal activity (Figure 2) does not involve membrane disruption, but has a different molecular target. Although considered rare in ant venoms, neurotoxic peptides are often found in animal venoms and their role is to achieve rapid prey immobilization [2]. These peptides typically block ion channels, with varying degrees of specificity and efficacy [2]. For example, the *P. clavata* peptide, poneratoxin, modulates voltage-gated sodium channels in both vertebrates and invertebrates, whereas the dimeric ectatomin Et-1 peptide from the neotropical ant, *Ectatomma tuberculatum*, blocks voltage-gated calcium channels and acts as a pore-forming peptide in eukaryotic cells [2,50]. Our behavioral assay revealed that crude *M. rubida* venom or the U-MYRTX-MANr1 peptide triggered severe paralysis after injection (Table 3 and Figure 3), with a much stronger effect than *M. rubra* venom components (Table S5 and Figure S5). This suggests that U-MYRTX-MANr1 may be a neurotoxin, although further studies are needed to confirm its activity against ion channels. 

In addition to paralysis, we observed significant body fluid loss in aphids injected with either crude *M. rubida* venom or the isolated peptide (Figure 3 and Figure S6). This indicates that hemolymph coagulation may be impaired, which is an important component of wound healing [60,61]. Venom components of the endoparasitoid wasp, *Pimpla turionellae* (Hymenoptera: Ichneumonidae), suppress hemocyte-mediated immune responses at the cellular level [62]. Therefore, further studies should examine the correlation between ant venom components and the inactivation of hemocytes and/or clotting factors that may be essential for wound healing in aphids. Furthermore, it would be interesting to investigate the observed cessation of aphid embryonic development and tissue alterations after exposure to *M. rubida* venom (Figure S6).

Although potent peptide toxins can be integrated directly into pest management strategies by spraying the toxins onto plants (e.g., Spear-T developed by Vestaron), the more typical approach is the development of insect-resistant transgenic crops or engineered entomopathogens (e.g., fungi or baculoviruses) [24,25]. The development of bio-insecticides based on venom peptides frequently fails due to their lack of stability or low oral activity, as shown for U-MYRTX-MANr1 [63]. Therefore, natural peptides are usually replaced with synthetic versions that are more stable, as with the *U. yaschenkoi* AMPs discussed above [58,64,65]. Chemical modifications such as the replacement of individual disulfide bonds with diselenide bonds can improve the oral activity of venom peptides, making them more suitable for the development of bio-insecticides [63]. 

In summary, the proteomic analysis of *M. rubida* venom and functional characterization of the novel peptide U-MYRTX-MANr1 make a significant contribution to the underrepresented field of ant venom research. Our insect bioassays suggest that the *M. rubida* peptide toxin may act simultaneously against several molecular targets (e.g., ion channels or hemolymph coagulation proteins) as previously shown for the ant venom toxin, ectatomin Et-1 [2]. The remarkable insecticidal activity of crude *M. rubida* venom indicates that additional peptide toxins could also be suitable as leads for the development of novel bio-insecticides.

## 4. Materials and Methods 

### 4.1. Ant Collection and Taxonomy

Ants identified as *M. rubida* workers based on their morphology, according to Seifert’s identification key [66], were collected from a sunny open habitat with a rocky-loamy soil in a forest >500 meters above sea level, located near Tambach-Dietharz, Thuringia, Germany. In the laboratory, foraging workers were kept in a plastic box (180 × 135 × 60 mm) containing soil and plant litter from the environment as an artificial nest, fitted with a test tube water reservoir (160 × 16 mm). Ants were provided with 20% sucrose solution and mealworms (*Tenebrio molitor*) twice a week and maintained under constant conditions (~23 °C, 40% relative humidity, and a 16 h photoperiod).

### 4.2. Crude Venom Extraction

*M. rubida* workers were anesthetized with CO_2_ and the sting apparatus was removed from the abdominal segment. Venom glands and reservoirs of five foraging workers were gently transferred to a 1.5 mL microcentrifuge tube and pooled in 100 µL of chilled methanol. The crude extract was immediately centrifuged for 30 min at 18,000× *g*. The supernatant was transferred to a new tube for crude venom analysis and fractionation. We used different solvents for crude venom extraction, for reduction and alkylation, and for injection. 

### 4.3. Reduction and Alkylation

Crude venom was extracted in 100 µL of 50 mM ammonium bicarbonate containing 5% acetonitrile and was supplemented with 5 mM dithiothreitol before incubation for 30 min at 56 °C. After cooling the mixture to room temperature, cysteine residues were alkylated with 15 mM iodoacetamide for 30 min in the dark.

### 4.4. LC-MS Analysis of Venom and Peptides

Conditions and settings for reversed-phase LC and MS data acquisition were modified slightly from our previous study [39]. Peptides were separated with 0.1% formic acid in water (eluent A) and 0.1% formic acid in acetonitrile (eluent B). The starting concentration (5% B) was maintained for 5 min, increased to 60% B in 60 min, then steeply increased to 95% B in 5 min, held for 9 min, then reduced to the starting concentration (5% B) in 1 min, followed by a re-equilibration step (10 min). The following MS instrument settings were used: capillary voltage 3200 V, Hexapole RF 200 Vpp, ion energy 5.0 eV, and collision RF 460 Vpp.

### 4.5. Edman Degradation

Automated N-terminal sequencing was performed by stepwise Edman degradation (Proteome Factory AG, Berlin, Germany) using a Procise Model 492 cLc protein sequencer (Applied Biosystems, Foster City, CA, USA) according to the manufacturer’s protocol.

### 4.6. Peptide Synthesis

The U-MYRTX-MANr1 peptide was synthesized by Romer Labs (Butzbach, Germany) according to the manufacturer’s protocol (purity > 98%).

### 4.7. Determination of Total Protein Concentration for Injection Assays

Peptides from six *M. rubida* venom glands or 12 *M. rubra* venom glands were extracted in 50 µL water as described above. Total protein concentration was determined using a NanoDrop 2000 spectrophotometer (Thermo Fisher Scientific, Waltham, MA, USA). The absorbance of a 1.5 µL droplet of crude venom was determined at 205 nm and the total protein concentration was calculated using an extinction coefficient (ε_205_) of 31 mL·mg^−1^·cm^−1^ [67] and Scopes’ method [68]. We used 1 mg·mL^−1^ solutions of BSA and the synthetic *M. rubra* decapeptide NH_2_-IDPKLLESLA-CONH_2_ [39] as controls. Crude extracts were further diluted to 1.0, 0.1 and 0.01 mg·mL^−1^ for aphid injection assays. 

### 4.8. Antimicrobial Assay

The minimal inhibition concentration (MIC) was determined by broth dilution assay in a 384-well plate with a final working volume of 20 µL as previously described [69]. Antibacterial and antifungal activity were screened against the following test strains: *Bacillus megaterium* ATCC 14945, *Bacillus subtilis* DSM 10, *Escherichia coli* D31, *Listeria fleischmannii* DSM 24998, *Listeria monocytogenes* ATCC 15313, *Micrococcus luteus* DSM 20030, Staphylococcus aureus ATCC 25923, *Moraxella catarrhalis* DSM 9143, *Pseudomonas aeruginosa* ATCC 27853, *Staphylococcus epidermidis* ATCC 35984, and *Candida albicans* ATCC 90028. We also used the synthetic analog of the *M. rubra* decapeptide U-MYRTX-MRArub1 [39]. Microbial strains were cultivated in Mueller–Hinton II or brain heart infusion broth, as appropriate. Peptides were serially diluted (two-fold), resulting in a concentration range from 512 to 0.016 µg·mL^−1^. Endpoint MIC values were read after 16 h of incubation at 37 °C (*C. albicans* 28 °C) with continuous shaking on a LUMIstar Omega plate reader (BMG Labtech, Ortenberg, Germany). We measured absorbance at 600 nm (*B. megaterium*, *B. subtilis*, *E. coli*, *P. aeruginosa* and *S. epidermidis*) or luminescence (*L. fleischmanii*, *L. monocytogenes*, *M. luteus*, *M. catarrhalis*, *S. aureus* and *C. albicans*) using BacTiter-Glo (Promega, Mannheim, Germany). All experiments were conducted in technical and biological triplicates, including sterility and growth controls (gentamycin and rifampicin).

### 4.9. Maintenance of Aphids, Injection and Incapacitation Assays

The parthenogenetic *A. pisum* clone LL01 was reared under constant conditions on broad beans, *Vicia faba* var. *minor*, as previously described [34,70]. Age-synchronized aphids (5 days old) were used in all experiments [71]. Aphids were injected laterally, between the middle and hind legs, with 25 nL of crude venom extract or peptides, using glass capillaries held on a M3301 micromanipulator (World Precision Instruments, Hitchin, UK). Crude *M. rubida* and *M. rubra* venoms were tested at concentrations of 0.01, 0.1 and 1 mg·mL^−1^, whereas peptide toxins U-MYRTX-MANr1 and U-MYRTX-MYRrub1 were tested at concentrations of 1, 4 and 16 mg·mL^−1^. The *M. rubra* peptide U-MYRTX-MYRrub1 was used as a control because its insecticidal activity had already been confirmed [39]. Water and BSA (1, 4 and 16 mg·mL^−1^) were used as negative controls. We injected 60 aphids in three biological replicates of 20 individuals per treatment. Injected aphids were reared individually for 10 days in Petri dishes with *V. faba* leaves on 1% agarose gel [71,72]. Aphid survival and offspring production were monitored daily [46]. Newly emerged nymphs were counted daily and removed. Fresh Petri dishes with *V. faba* leaves were provided every 5 days to ensure the aphids were maintained in an optimal environment.

Aphid behavior was observed after injections with crude venom (1 mg·mL^−1^) or the pure peptides (16 mg·mL^−1^) compared to a water control, with 30 individuals injected per treatment group. Injected aphids were placed on their backs and the time they took to correct their position was recorded [4]. Aphid fitness and behavior were assessed immediately after and 1 h after injection and images were acquired using a Leica M125 C stereomicroscope. 

### 4.10. Feeding Assays and Insecticide Bioassays

The oral activity of toxin U-MYRTX-MANr1 and its effect on aphid susceptibility to chemical insecticides were tested as previously described [39] with minor modifications. Briefly, we fed *A. pisum* nymphs (5 days old) for 3 days in modified chambers [73] on an artificial diet [74] mixed with U-MYRTX-MANr1 (500 µg·mL^−1^) or a negative control diet in which the peptide solution was replaced with water. Survival was scored daily over the 3 days of feeding. We tested ~1300 aphids per treatment in three biological replicates. 

Aphids that survived the 3 day feeding treatment with U-MYRTX-MANr1 or the control were transferred to insecticide bioassays. We tested three chemical insecticides: imidacloprid (neonicotinoid), methomyl (carbamate) and spirotetramat (tetramic acid derivative) [18,32]. All were acquired from Chem Service Inc. (West Chester, PA, USA). For each insecticide, a stock solution of 1000 µg·mL^−1^ was prepared in acetone and working solutions (imidacloprid = 0.0975 µg·mL^−1^; methomyl = 6.25 µg·mL^−1^; spirotetramat = 1.56 µg·mL^−1^) were prepared in water. Sub-lethal concentrations of each compound were used, based on previous studies [46]. 

Insecticide bioassays were carried out as previously described [39,46]. Briefly, *V. faba* stems with roots (2–3 weeks old) were dipped into Falcon tubes (50 mL) containing each insecticide working solution for 24 h. Petri dishes were then prepared with bean leaf discs prepared from the treated stems, as recommended by the Insecticide Resistance Action Committee (IRAC) [75]. Ten aphids, previously treated with the peptide or control diet, were transferred to each leaf disc in 6–8 replicates for each insecticide. Each experiment was conducted with three biological replicates, along with corresponding solvent and water controls. Aphid mortality was scored after exposure for 3 days. 

### 4.11. Histological Preparations

For light microscopy, 30 aphids (5 days old) 12 h after each treatment were pre-fixed in 2.5% glutaraldehyde in 0.1 M phosphate buffer (pH 7.4) for 1 h. Treatment groups were assigned as follows: (I) water injection, (II) peptide toxin U-MYRTX-MANr1 (16 mg·mL^−1^), and (III) crude *M. rubida* venom (1 mg·mL^−1^). After washing in phosphate buffer, aphids were post-fixed in 1% OsO_4_ in the same buffer for 1 h. After dehydration in a graded ethanol series, aphids were embedded in Araldite epoxy resin (Plano, Germany). Semi-thin sections were prepared from five randomly chosen specimens representing each treatment using a Reichert Om/U3 ultramicrotome. Sections were stained with 1% toluidine blue in 1% sodium borate and examined using a Leica DM 4 B microscope.

### 4.12. Data Analysis

Molecular features were extracted from acquired mass spectra using Compass DataAnalysis v4.2 (Bruker Daltonics, Billerica, MA, USA) as previously described [39]. MS data were analyzed and visualized using SigmaPlot v12.5 (Systat Software, San Jose, CA, USA). We analyzed aphid fitness data using IBM SPSS Statistics v17 (Armonk, New York, NY, USA). Statistical significance was defined as *p* < 0.05. Survival data were analyzed by Kaplan–Meier survival analysis and comparisons between groups were based on log-rank tests. The total number of offspring was analyzed using the Mann–Whitney *U* test for non-parametric data and Student’s t-test for normally distributed data. For insecticide bioassays, total mortality for each insecticide treatment was corrected according to Abbott’s formula, based on mortality scored in control groups [76]. The mortality in solvent control groups was 4–12%. We used the Mann–Whitney *U* test to compare mortality between the two feeding treatments (peptide toxin and diet control). For sequence alignment, we used Clustal Omega v1.2.4 from the European Bioinformatics Institute (Hinxton, UK) [77] and the online tool Sequence Identity And Similarity (SIAS) [78].

## Figures and Tables

**Table 1:** The most abundant peptides (relative peak intensity ≥ 3%) in the venom of *Manica rubida*. The venom peptidome is dominated by linear peptides, but contains a few peptides with one intramolecular disulfide bond. RT = retention time, Int. = relative peak intensity, MW = molecular weight, S-S = disulfide bond.

No.	RT [min]	Int. [%]	MW_crude_ [Da]	MW_red._ [Da]	MW_alk._ [Da]	S-S	Length ^a^
1	26.3	8	1314.71	1314.71	1314.72	0	12
2	26.3	7	1352.66	1352.66	1352.66	0	12
3	26.3	4	1336.69	1336.70	1336.70	0	12
4	27.0	77	1434.80	1436.83	1550.87	1	13
5	27.1	8	1208.64	1210.66	1324.70	1	11
6	27.1	3	1472.75	1474.77	1588.81	1	13
7	32.1	15	1132.64	1132.65	1132.65	0	10
8	32.1	28	1093.64	1093.64	1093.64	0	10
9	32.1	17	1148.61	1148.61	1148.61	0	10
10	32.1	7	920.50	920.50	920.50	0	8
11	32.1	4	994.57	994.57	994.57	0	9
12	32.1	100	1110.67	1110.68	1110.66	0	10
13	38.9	4	1136.68	1136.68	1136.68	0	10
14	43.4	62	2739.63	2739.64	2739.64	0	25
15	43.4	12	2569.52	2569.53	2569.53	0	23
16	43.6	17	2978.60	2978.61	2978.61	0	27
17	44.7	5	2850.72	2850.72	2850.73	0	26
18	46.3	3	2987.79	2987.79	2987.79	0	27
19	51.5	3	2823.76	2823.77	2823.77	0	25
20	51.5	19	2840.79	2840.80	2840.80	0	26
21	54.5	92	2174.27	2174.27	2174.28	0	20
22	54.6	3	2218.22	2218.23	2218.23	0	20
23	54.6	45	2196.24	2196.25	2196.25	0	20
24	54.6	18	2212.21	2212.21	2212.21	0	20
25	54.6	4	2042.10	2042.11	2042.11	0	18
26	55.2	7	2840.79	2840.80	2840.80	0	26

^a^ The amino acid sequence length was calculated using averagine (111.1254) [44].

**Table 2:** Crude ant venoms and associated peptidyl toxins strongly reduced the survival of injected aphids (*Acyrthosiphon pisum*) (60 aphids in three biological replicates of 20 individuals per treatment).

Treatment		Concentration (mg·mL^−1^)	% Survival	Significance ^a^
Bovine serum albumin (BSA) ^b^		16	78.3	ns
		4	84.7	ns
		1	91.7	*p* < 0.05
Crude venom	*Manica rubida*	1	0.0	*p* < 0.0001
		0.1	33.3	*p* < 0.0001
		0.01	66.7	ns
	*Myrmica rubra*	1	20.0	*p* < 0.0001
		0.1	71.7	ns
		0.01	66.7	ns
Peptides	U-MYRTX-MANr1 (*Manica rubida*)	16	6.7	*p* < 0.0001
		4	30.0	*p* < 0.0001
		1	75.0	ns
	U-MYRTX-MYRrub1 (*Myrmica rubra*)	16	41.7	*p* < 0.0001
		4	60.0	*p* < 0.01
		1	80.0	ns

^a^ Compared to water (survival ~78%, 10 days post-injection); ^b^ BSA was used as peptide/protein control; ns = not significant.

**Table 3:** Aphid behavior and fitness after the injection of water, U-MYRTX-MANr1 (16 mg·mL^−1^) or *Manica rubida* crude venom (1 mg·mL^−1^) (30 individuals injected per treatment). Observations were recorded immediately after injection and 1 h later.

Treatment	Immediately Post-Injection	1 h Post-Injection
Control (water)	Immediate righting; responsiveness < 10 s; fast and active movement	No visible impact on vitality or fitness
U-MYRTX-MANr1 16 mg·mL^−1^	Delayed righting Response; ~1 min; Disoriented and slow movement (mild paralysis)	Moderate reduction of vitality and fitness; continuous mild paralysis
Crude venom 1 mg·mL^−1^	Delayed or no righting (>3 min); very limited movement (strong paralysis)	Extreme reduction of vitality and fitness (~40% with no physiological reactions); continuous strong paralysis; necrosis and extensive loss of hemolymph

## Supplementary Materials

The following are available online at https://www.mdpi.com/2072-6651/11/10/562/s1, Figure S1: Distribution of peptides in the venom of *Manica rubida* (*n* = 96 peptides) according to amino acid sequence length and molecular weight. Figure S2: Fragmentation pattern of the peptide U-MYRTX-MANr1. Table S1: Effect of crude venom and peptide toxins on the total number of offspring in *Acyrthosiphon pisum*. Table S2: Statistical data for the Kaplan–Meier survival analysis shown in Figure 2. Figure S3: Insecticidal activity of *Myrmica rubra* crude venom and peptide U-MYRTX-MRArub1 in *Acyrthosiphon pisum.* Table S3: Statistical data for Kaplan–Meier survival analysis shown in Figure S3. Figure S4: Insecticidal activity of bovine serum albumin (BSA) control in *Acyrthosiphon pisum*. Table S4: Statistical data for Kaplan–Meier survival analysis shown in Figure S4. Table S5: Aphid behavior and fitness after the injection of water, U-MYRTX-MYRrub1, or *Myrmica rubra* crude venom. Figure S5: Injection wound healing in *Acyrthosiphon pisum* 1 h post-injection of water, U-MYRTX-MYRrub1, or crude *Myrmica rubra* venom. Figure S6: Semi-thin cross-section through the abdomen of *Acyrthosiphon pisum* injected with crude *Manica rubida* venom. Figure S7: Semi-thin cross-section through abdomen of *Acyrthosiphon pisum* injected with *Manica rubida* peptide U-MYRTX-MANr1. Figure S8: Semi-thin cross-section through the abdomen of *Acyrthosiphon pisum* injected with water. Figure S9: Insecticidal activity of orally delivered *Manica rubida* peptide U-MYRTX-MANr1. Table S6: Summary of statistical data for the insecticidal activity of orally delivered *Manica rubida* peptide U-MYRTX-MANr1 and its effect on the susceptibility of aphids to chemical insecticides. Figure S10: Alignment of U-MYRTX-MANr1 with peptide sequences found in the venom of other arthropods or defensive skin secretions of frogs.
