# Supplementary material for: Identification and Functional Characterization of a Novel Insecticidal Decapeptide from the Myrmicine Ant Manica rubida
Source: Toxins (Basel). 2019 Sep 25;11(10):562. doi: 10.3390/toxins11100562 (PMC6832575; doi:10.3390/toxins11100562)
Supplement: Supplementary file 1 [file toxins-11-00562-s001.pdf]

Supplementary Materials: Identification and Functional Characterization of a Novel Insecticidal Decapeptide from the Myrmicine Ant *Manica rubida*

John Heep, Marisa Skaljac, Jens Grotmann, Tobias Kessel, Maximilian Seip, Henrike Schmidtberg and Andreas Vilcinskas

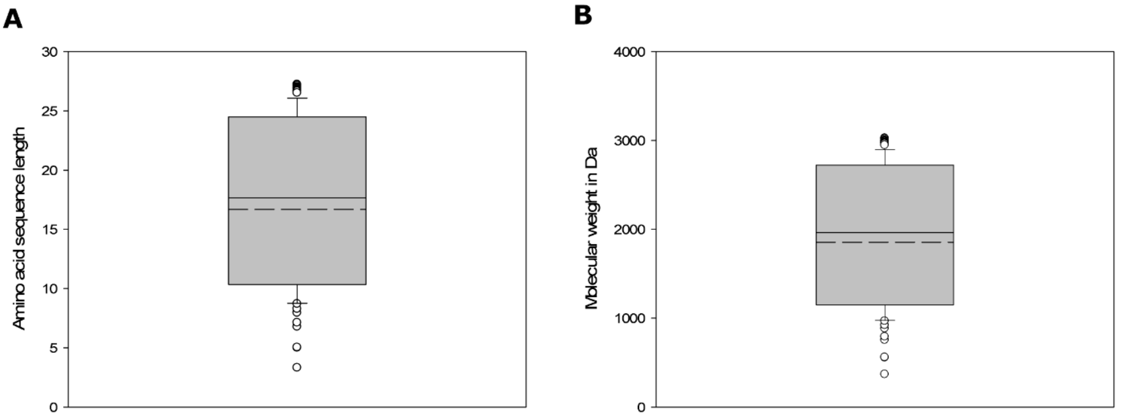

Figure S1. The distribution of peptides in the venom of *Manica rubida* (n = 96 peptides) according to amino acid sequence length (**A**) and molecular weight (**B**) displayed as box-and-whisker plots. The amino acid sequence length was predicted using averagine (111.1254 Da) [1]. Boundaries = 25th and 75th percentiles, whiskers = 10th and 90th percentiles, median = solid line, and mean = dashed line.

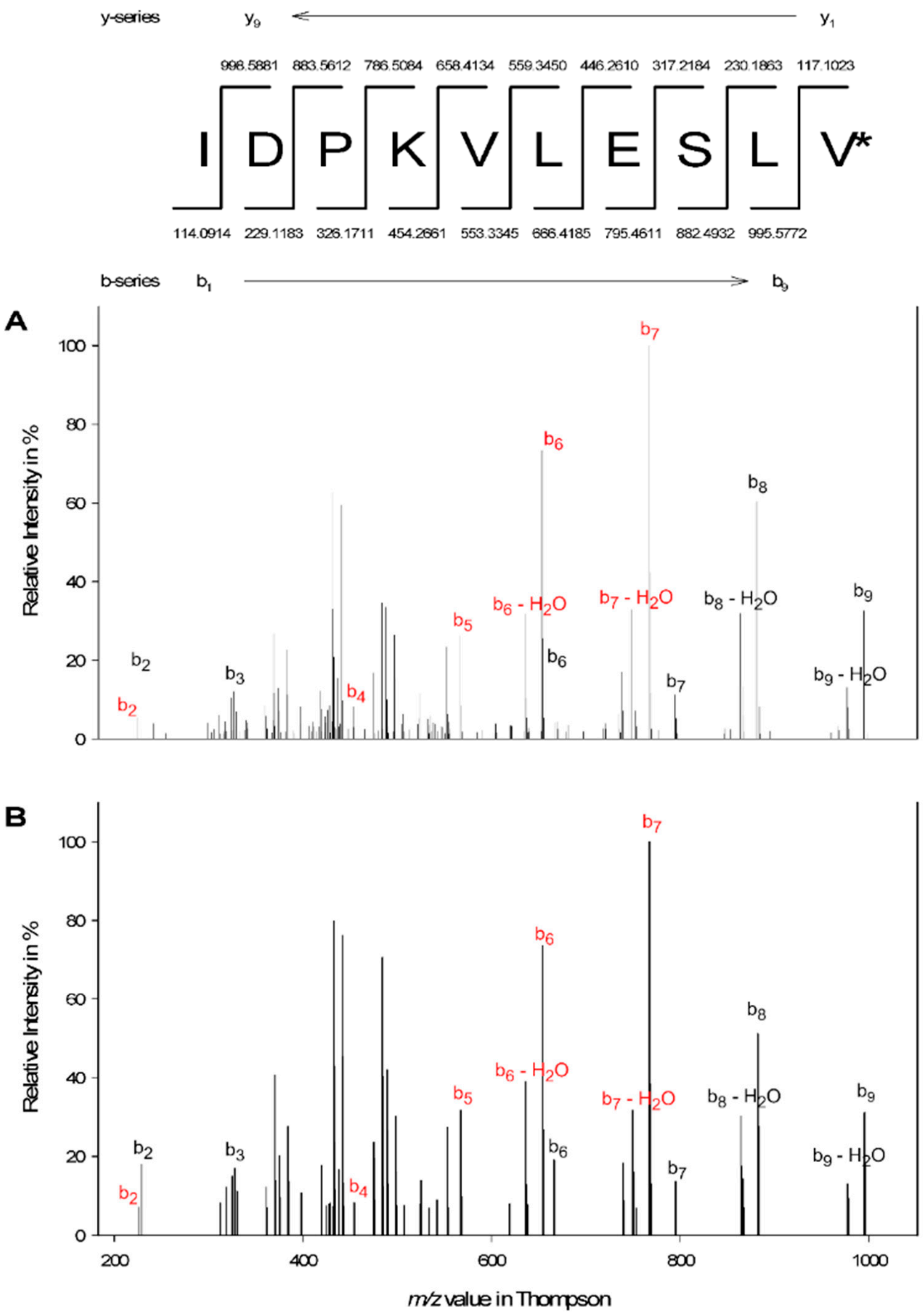

Figure S2. The fragmentation pattern of the peptide U-MYRTX-MANr1 showed high congruency in its native form (**A**) compared to its synthetic analog (**B**). The spectrum is dominated by fragments of the *b*-series ions of both the peptide itself (black) and the internal cleavage fragment PKVLESLV (red). The asterisk indicates C-terminal amidation. The spectra were acquired by collision-induced dissociation (25 eV) with nitrogen gas on a mircOTOF-QII instrument (Bruker, USA).

Table S1. Effects of the crude venom and peptide toxins on the total number of offspring in *Acyrthosiphon pisum* (60 aphids in three biological replicates of 20 individuals per treatment).

	Treatment	Concentration ($mg \cdot mL^{-1}$)	Total Number of Offspring §	Significance ^
Control	Water	/	43.31 ± 1.40	/
	Bovine serum albumin (BSA)	16	43.67 ± 1.43	ns
		4	42.96 ± 1.87	ns
		1	48.50 ± 1.85	$p < 0.05$
Crude venom	*Manica rubida*	0.1	32.15 ± 3.04	$p < 0.05$
		0.01	38.44 ± 2.57	ns
	Myrmica rubra	0.1	40.87 ± 1.83	ns
		0.01	38.36 ± 2.08	ns
Peptides	*Manica rubida* (U-MYRTX-MANr1)	4	31.80 ± 3.78	$p < 0.01$
		1	39.54 ± 1.95	ns
	Myrmica rubra (U-MYRTX-MRArub1)	4	37.74 ± 2.89	ns
		1	42.56 ± 1.94	ns

§ Mean ± standard error (SE). ^ Compared to water. Significance: (/) = not applicable; (ns) = not significant ($p > 0.05$).

Table S2. Statistical data for Kaplan-Meier survival analysis (60 aphids in three biological replicates of 20 individuals per treatment), as shown in Figure 2.

Treatment	Mean§				Significance (Water vs. Treatment)
	Estimate	Standard Error	95% Confidence Interval		
			Lower Bound	Upper Bound	
Water	8.78	0.31	8.17	9.39	/
Crude venom (*Manica rubida*) (1 $mg \cdot mL^{-1}$)	1.00	0.00	1.00	1.00	$p < 0.0001$
Crude venom (*Manica rubida*) (0.1 $mg \cdot mL^{-1}$)	6.22	0.39	5.45	6.98	$p < 0.0001$
Crude venom (*Manica rubida*) (0.01 $mg \cdot mL^{-1}$)	8.30	0.38	7.56	9.04	ns
Water	9.15	0.19	8.78	9.52	/
U-MYRTX-MANr1 (*Manica rubida*) (16 $mg \cdot mL^{-1}$)	3.43	0.34	2.78	4.09	$p < 0.0001$
U-MYRTX-MANr1 (*Manica rubida*) (4 $mg \cdot mL^{-1}$)	5.61	0.47	4.71	6.53	$p < 0.0001$
U-MYRTX-MANr1 (*Manica rubida*) (1 $mg \cdot mL^{-1}$)	9.03	0.28	8.48	9.59	ns

§ Estimate is limited to the largest survival time if it is censored. Significance: (/) = not applicable; (ns) = not significant ($p > 0.05$).

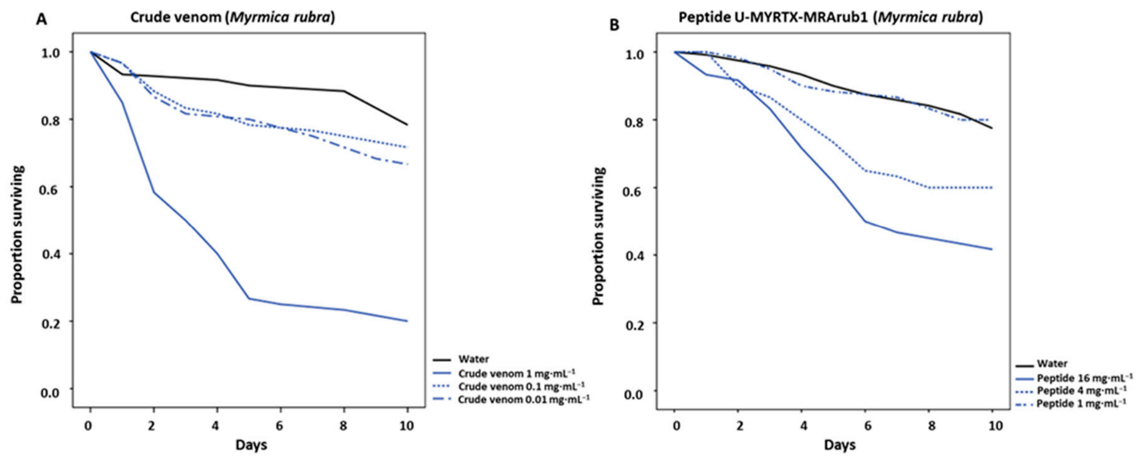

Figure S3. Low aphid survival reveals substantial insecticidal activity (~80% mortality) for crude *Myrmica rubra* venom at the highest concentration (1 mg·mL^{-1}), whereas high (16 mg·mL^{-1}) and medium (4 mg·mL^{-1}) concentrations of the peptide U-MYRTX-MRArub1 induced significant mortality of ~58% and ~40%, respectively. Survival (60 aphids in three biological replicates of 20 individuals per treatment) was monitored for 10 days after the injection of crude venom (**A**) or the peptide toxin (**B**) into pea aphids, *Acyrthosiphon pisum*. Survival data were analyzed using Kaplan-Meier statistics and comparisons between the treatment and control were based on log-rank tests. Statistical data are shown in Tables 2 and S3.

Table S3. Statistical data for Kaplan-Meier survival analysis (60 aphids in three biological replicates of 20 individuals per treatment), as shown in Figure S3.

**Treatment**	**Mean** §				**Significance (Water vs. Treatment)**
	**Estimate**	**Standard Error**	**95% Confidence Interval**		
			**Lower Bound**	**Upper Bound**	
Water	9.13	0.32	8.50	9.77	/
Crude venom (*Myrmica rubra*) (1 mg·mL^{-1})	4.57	0.43	3.72	5.42	$p < 0.0001$
Crude venom (*Myrmica rubra*) (0.1 mg·mL^{-1})	8.32	0.41	7.52	9.11	ns
Crude venom (*Myrmica rubra*) (0.01 mg·mL^{-1})	8.22	0.41	7.42	9.01	ns
Water	9.15	0.19	8.78	9.52	/
U-MYRTX-MRArub1 (*Myrmica rubra*) (16 mg·mL^{-1})	6.88	0.41	6.08	7.68	$p < 0.0001$
U-MYRTX-MRArub1 (*Myrmica rubra*) (4 mg·mL^{-1})	7.78	0.38	7.03	8.53	$p < 0.01$
U-MYRTX-MRArub1 (*Myrmica rubra*) (1 mg·mL^{-1})	9.10	0.27	8.57	9.63	ns

§ Estimate is limited to the largest survival time if it is censored. Significance: (/) = not applicable; (ns) = not significant ($p > 0.05$).

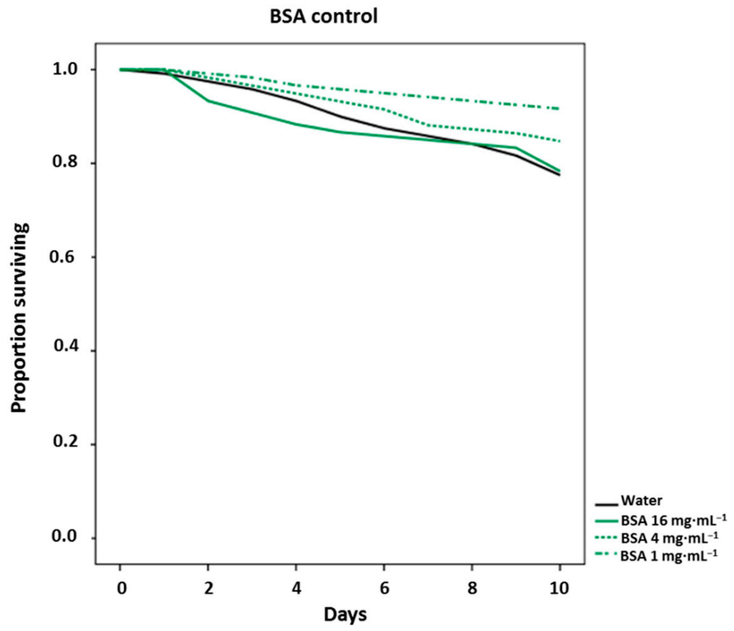

Figure S4. Bovine serum albumin (BSA) treatment did not significantly impact survival, except for the low concentration (1 mg·mL^{-1}) which improved survival ($p < 0.05$) compared to the control treatment (diet diluted with water). Survival (60 aphids in three biological replicates of 20 individuals per treatment) was monitored for 10 days after the injection of BSA into pea aphids, *Acyrthosiphon pisum*. Survival data were analyzed using Kaplan-Meier statistics and comparisons between the treatment and control were based on log-rank tests. Statistical data are shown in Tables 2 and S4.

Table S4. Statistical data for Kaplan-Meier survival analysis (60 aphids in three biological replicates of 20 individuals per treatment), as shown in Figure S4.

Treatment	Mean §				Significance (Water vs. Treatment)
	Estimate	Standard Error	95% Confidence Interval		
			Lower Bound	Upper Bound	
Water	9.15	0.19	8.78	9.52	/
BSA (16 mg·mL^{-1})	9.02	0.32	8.39	9.65	ns
BSA (4 mg·mL^{-1})	9.37	0.25	8.89	9.85	ns
BSA (1 mg·mL^{-1})	9.72	0.81	9.36	10.08	$p < 0.05$

§ Estimate is limited to the largest survival time if it is censored. Significance: (/) = not applicable; (ns) = not significant ($p > 0.05$).

Table S5. Aphid behavior and fitness after injection of water, U-MYRTX-MYRrub1 (16 mg·mL^{-1}), or *Myrmica rubra* crude venom (1 mg·mL^{-1}) (30 individuals injected per treatment). Observations were recorded immediately and 1 h later.

**Treatment**	**Immediately Post-Injection**	**1 h Post-Injection**
Control<br>(water)	Immediate righting;<br>responsiveness < 10 s;<br>fast and active movement	No visible impact on<br>vitality and fitness
U-MYRTX-<br>MYRrub1<br>16 mg·mL^{-1}	Immediate righting;<br>responsiveness < 10 s;<br>slow and hesitant movement	Mild reduction of vitality<br>and fitness (mild paralysis)
Crude venom<br>1 mg·mL^{-1}	Mildly delayed correction of position;<br>responsiveness 10–30 s; slow-moving<br>(moderate paralysis)	Moderate reduction of<br>vitality and fitness;<br>continuous mild paralysis<br>and disturbed wound<br>healing

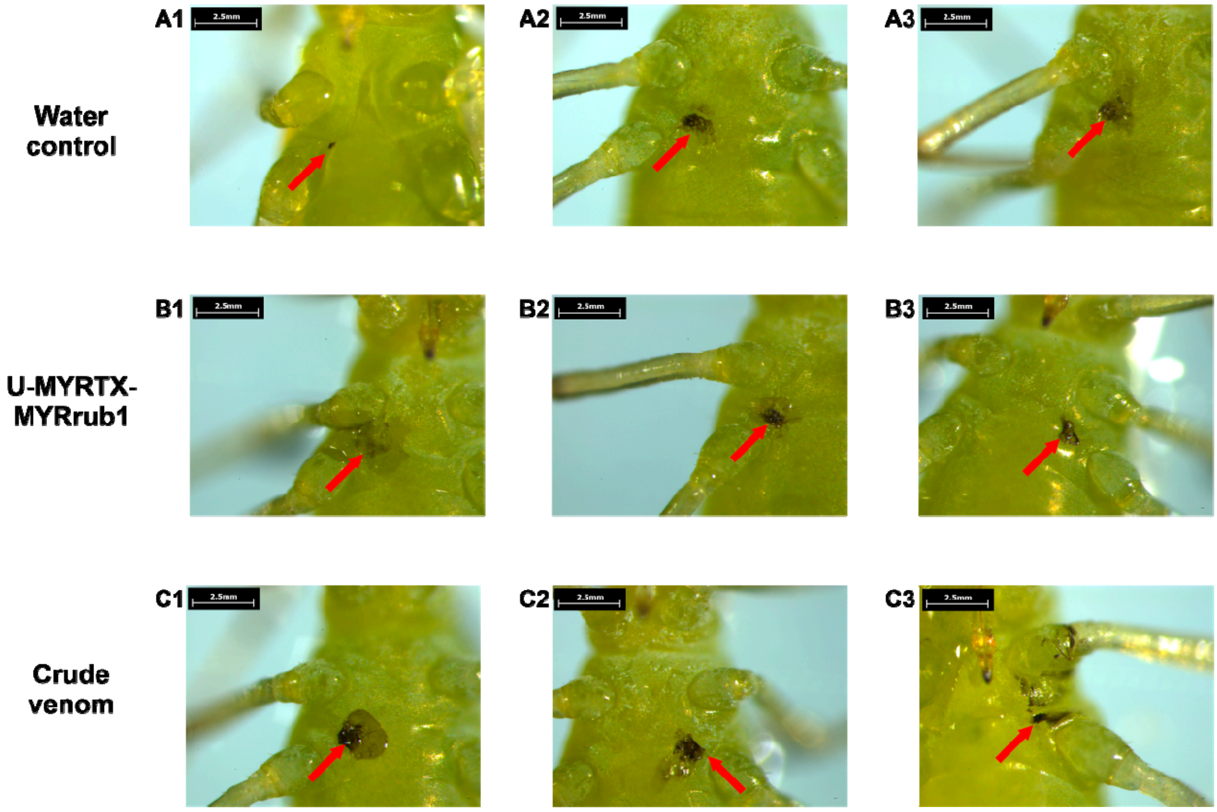

Figure S5. Injection wound healing in *Acyrthosiphon pisum* 1 h post-injection of water (control, A1-A3), U-MYRTX-MYRrub1 (16 mg·mL^{-1}, B1-B3), or crude *Myrmica rubra* venom (1 mg·mL^{-1}, C1-C3) (30 individuals injected per treatment). For aphids injected with U-MYRTX-MYRrub1, the injection site showed mild deterioration in hemolymph coagulation, whereas crude venom impaired wound healing, resulting in a moderate loss of hemolymph in the treated aphids. Injection sites and melanization of the exposed hemolymph are indicated with red arrows.

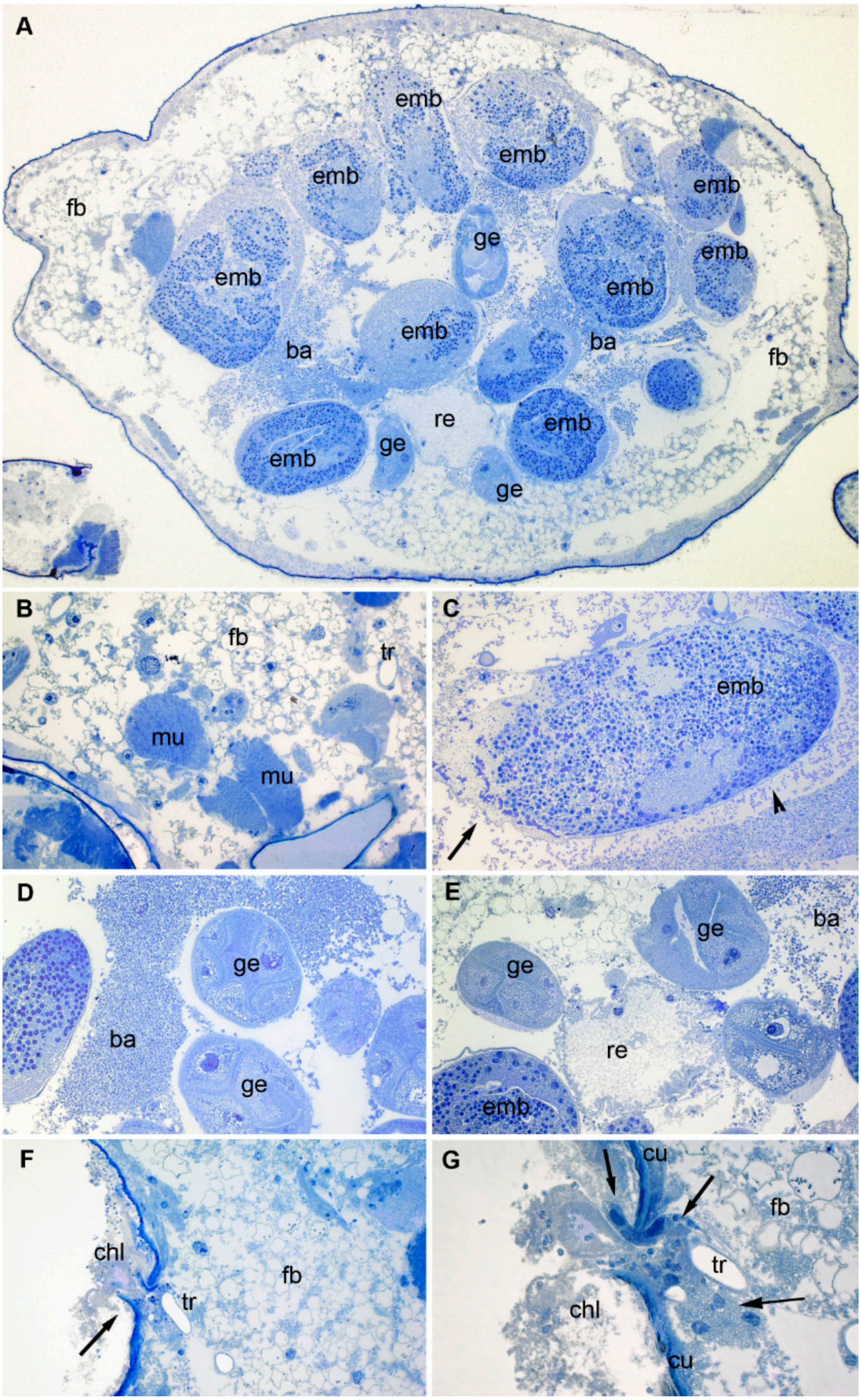
A
emb
emb
emb
emb
emb
emb
emb
emb
emb
emb
fb
fb
ge
ge
ge
ba
ba
re
B
fb
tr
mu
mu
C
emb
D
ge
ge
ba
E
ge
ge
ba
re
emb
F
chl
tr
fb
G
cu
cu
fb
tr
chl

Figure S6. Semi-thin cross-sections through the abdomens of *Acyrthosiphon pisum* (30 aphids) injected with crude *Manica rubida* venom (1 mg·mL^{-1}). (**A**) All embryos are arrested in early developmental stages, indicating retarded development. (**B**) The fat body tissue is in an advanced state of decomposition. Large parts of the fat body have degenerated and instead diminutive granular tissue is widely distributed and particularly dense in close proximity to the embryos (**C**, arrowhead). (**D**) Bacteriocyte clusters are dissociated and bacteria are often distributed between the embryos (**E**). (**F**) The injection site (arrow) shows impaired wound healing, resulting in a considerable loss of hemolymph, which is trapped externally and the opening of the still damaged cuticle. (**G**) Higher magnification of the injection site reveals hemocytes with dense cytoplasm that are located in (bold arrows) and close (arrow) to the open cuticle. Abbreviations: ba, bacteria; bc, bacteriocyte; chl, clotted hemolymph; cu, cuticle; dfb, dark fat body; emb, embryo; fb, fat body; ge, germarium; hg, hindgut; mg, midgut; mu, muscle; re, rectum; sm, serosa membrane; tr, tracheole; wa, wing anlagen.

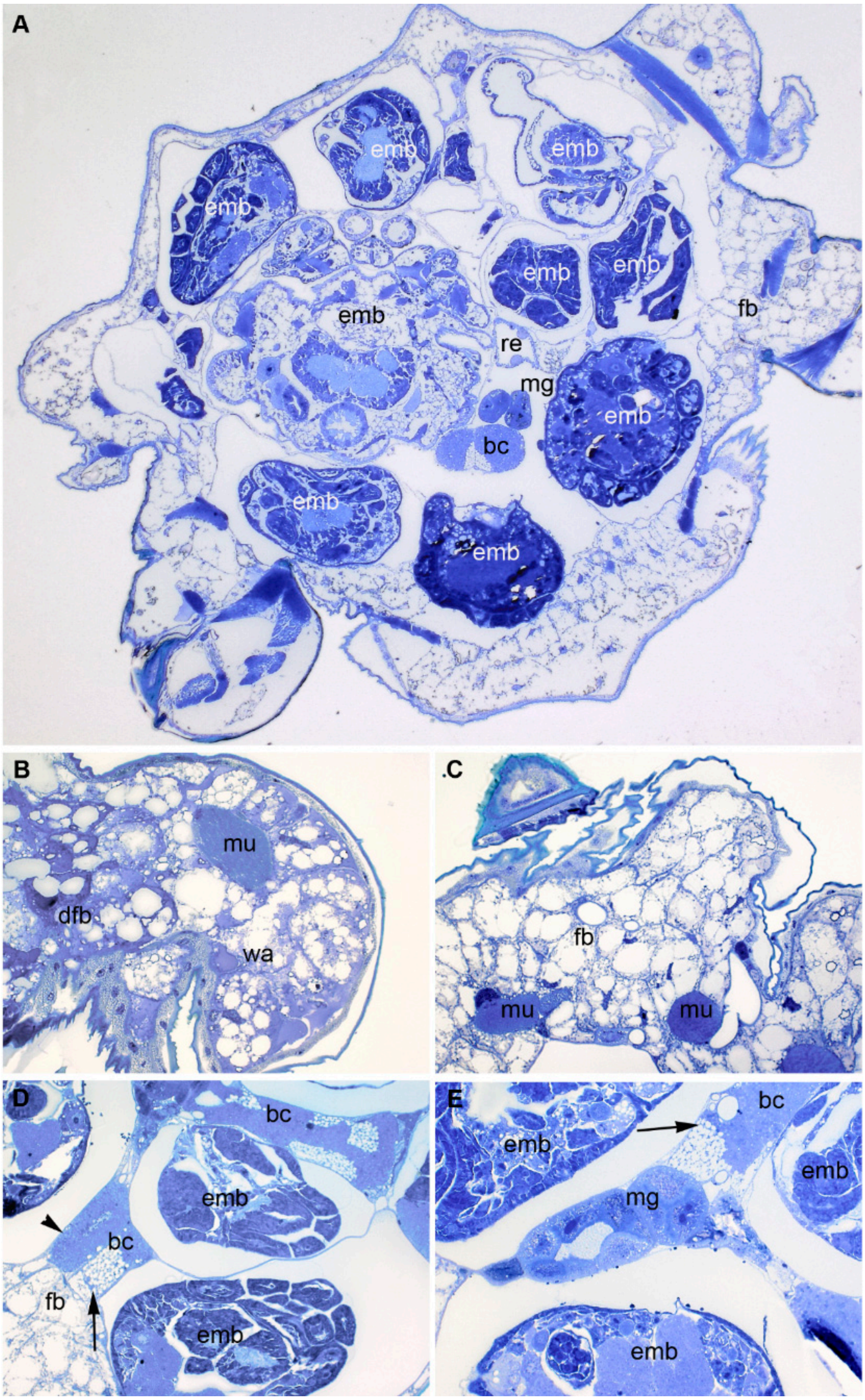
A
emb
emb
emb
emb
emb
emb
fb
re
mg
emb
bc
emb
emb
B
mu
dfb
wa
C
fb
mu
mu
D
bc
emb
bc
fb
emb
E
bc
emb
emb
mg
emb

Figure S7. Semi-thin cross-sections through abdomens of *Acyrthosiphon pisum* (30 aphids) injected with *Manica rubida* peptide U-MYRTX-MANr1 (16 mg·mL^{-1}). (**A**) Peptide treatment did not induce morphological differences compared to the control treatment. (**B**) and (**C**) The fat body tissue of both cell types is well organized and show no deterioration because of peptide treatment. (**D**) Bacteriocytes show vacuole dense zones (arrow) and symbiont-dense zones (arrowhead) as representative for adult individuals. (**E**) The midgut lies in close proximity to the bacteriocytes (arrow). In our histological analysis, we could not detect the injection site morphologically. For abbreviations see Figure S6.

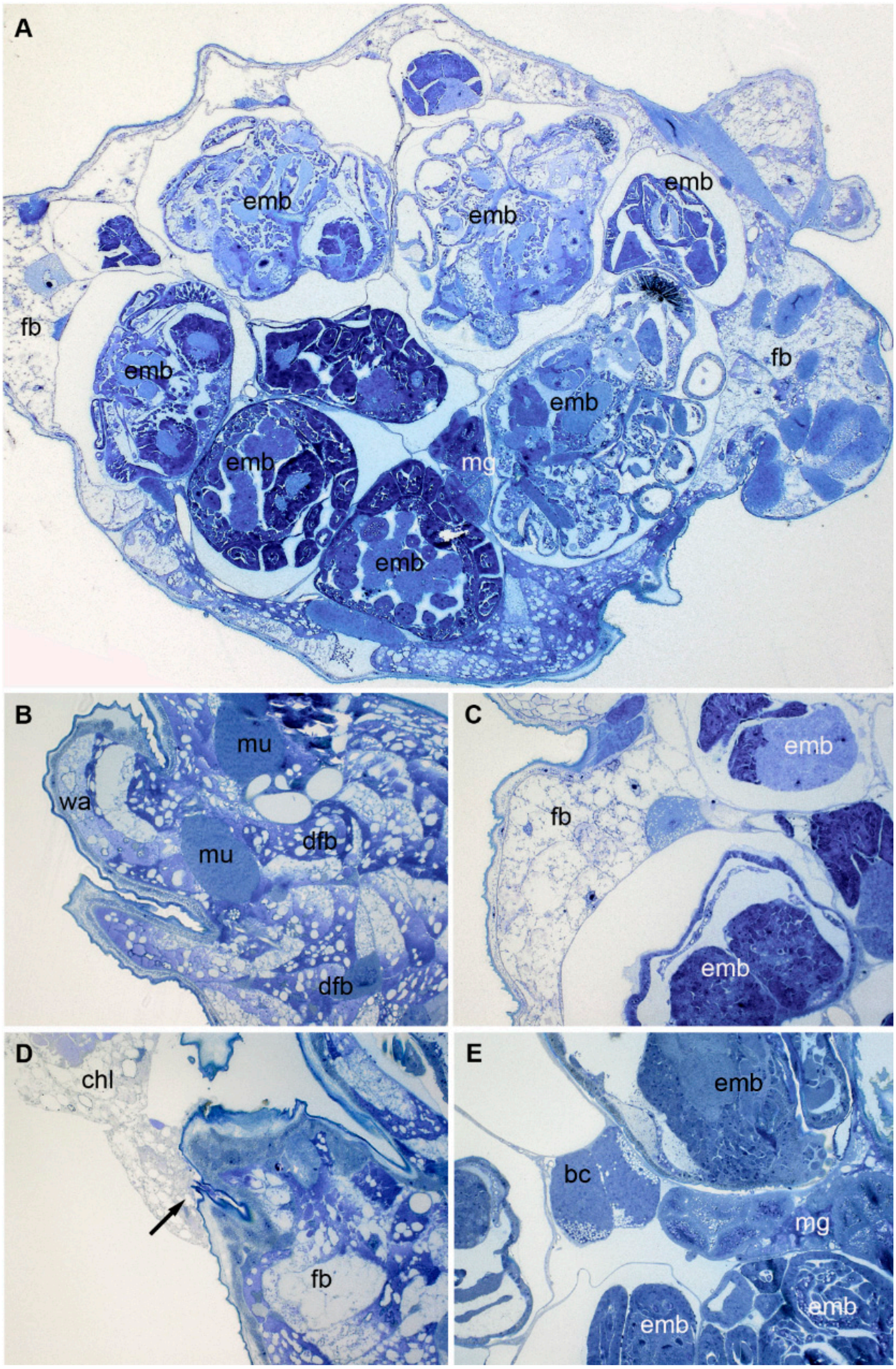

Figure S8. Semi-thin cross-sections through the abdomens of *Acyrthosiphon pisum* (30 aphids) injected with water (control treatment). (**A**) Embryos of all stages are present in the abdomen and enclosed by the serosa and the epithelium of the ovariole, which are often detached from the embryo due to the fixatives. The fat

body is formed of two types of cells with different cytoplasmic density. (**B)** In parts of the wing formation (wing anlagen), "dark" fat body cells with dense cytoplasm are prevalent. (**C**) At the caudal end of the abdomen, lucid fat body tissue replaces the dense form. (**D**) In some individuals, the injection site (arrow) is still visible because of the externally coagulated hemolymph, but wound healing has resulted in an intact cuticle. (**E**) Bacteriocytes are located in proximity to the gut. For abbreviations, see Figure S6.

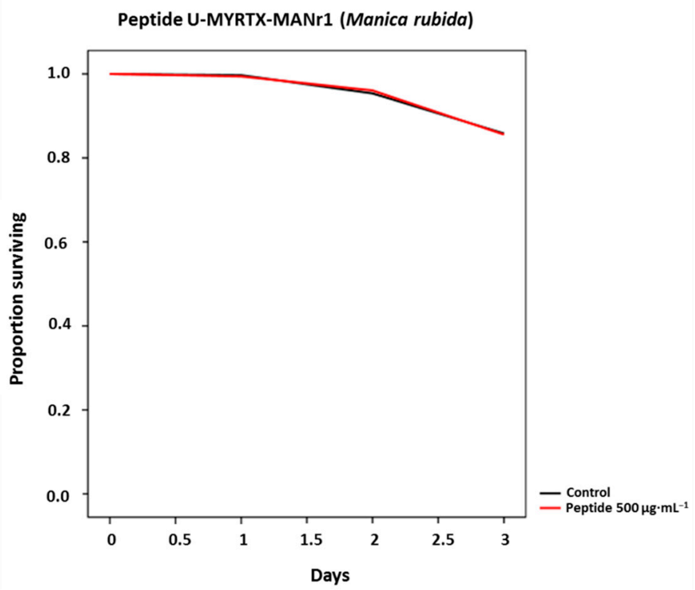

Figure S9. Insecticidal activity of orally delivered *Manica rubida* peptide U-MYRTX-MANr1. *Acyrthosiphon pisum* survival (~1300 aphids per treatment in three biological replicates) was monitored for 3 days of feeding on a specialized diet mixed with the test peptide (500 µg·mL^{-1}). Survival data were evaluated using Kaplan-Meier statistics and comparisons between the two groups were based on log-rank tests. Statistical data are shown in Table S6. The peptide treatment did not induce significant aphid mortality compared to the control treatment (diet diluted with water).

Table S6. Summary of statistical data for the insecticidal activity of orally delivered *Manica rubida* peptide U-MYRTX-MANr1 and its effect on the susceptibility of aphids (60–80 aphids per treatment in three biological replicates) to chemical insecticides.

**Treatment**	**Survival During the 3 days Feeding on a Specialized Diet [2] Alone or Mixed with the Peptide (See Figure S9)**				**Significance (Control vs. Peptide)**
	**Mean**				
	**Estimate**	**Standard Error**	**95% Confidence Interval**		
			**Lower Bound**	**Upper Bound**	
Control	2.95	0.006	2.94	2.96	/
U-MYRTX-MANr1 (*Manica rubida*) (500 µg·mL^{-1})	2.96	0.006	2.94	2.97	ns
Chemical insecticides (concentration)	Treatment/mortality§ after exposure to chemical insecticides				Significance
	Control		U-MYRTX-MANr1 (*Manica rubida*)		
Imidacloprid (0.0975 µg·mL^{-1})	77.43 ± 2.85		70.26 ± 4.54		ns
Spirotetramat (1.56 µg·mL^{-1})	48.91 ± 4.48		45.66 ± 4.45		ns
Methomyl (6.25 µg·mL^{-1})	57.29 ± 6.51		69.22 ± 5.34		ns

§Mean ± SE; ns = not significant ($p > 0.05$).

**Peptide**	**Source**	**Alignment**	**aa**	**MW [Da]**	**I%**	**S%**	**HP%**	**nc**	**Ref.**
U-MYRTX-MANr1	*Manica rubida* (ant)	- - - - - - - - - - - - - - - I D P K V L E S L V - - - - - - - - *	10	1111.33	100	100	60	0	this study
U-MYRTX-MRArub1	*Myrmica rubra* (ant)	- - - - - - - - - - - - - - - I D P K L L E S L A - - - - - - - - *	10	1097.31	80	100	60	0	Heep et al. (2019)
U$_{12}$-MYRTX-Tb1a	*Tetramorium bicarinatum* (ant)	- - - - - - - - - - - - - - - L S P A V L A S L A - - - - - - - - *	10	940.14	50	70	90	1	Touchard et al. (2018)
Temporin-H	*Rana temporaria* (frog)	- - - - - - - - - - - - - - - L S P N L L K S L L - - - - - - - - *	10	1096.36	40	70	60	2	Simmaco et al. (1996)
Temporin-A	*Rana temporaria* (frog)	- - - - - - - - - - - - F L P L I G R V L S G I L - - - - - - - - *	13	1396.76	15	38	85	2	Simmaco et al. (1996)
Temporin-1Vb	*Rana virgatipes* (frog)	- - - - - - - - - - - - F L S I I A K V L G S L F - - - - - - - - *	13	1406.75	8	38	77	2	Conlon et al. (2005)
VCT-VT1	*Vespa tropica* (wasp)	- - - - - - - - - - - - F L P I I G K L L S G L L - - - - - - - -	13	1383.76	15	38	85	1	Yang et al. (2013)
U-PONTX-Dq1b	*Dinoponera quadriceps* (ant)	- - - - - - - - - - - - L P L D D L S - - - - - D T - - - - - -	9	988.05	10	30	44	-3	Cologna et al. (2013)
M-PONTX-Ng1e	*Neoponera goeldii* (ant)	- - - - F W G A L I K G A A K L I P S V V G L F K K K Q - - - - -	24	2600.19	0	25	71	5	Orivel et al. (2001)
U-PONTX-Da2a	*Dinoponera australis* (ant)	- - - - F L G G L I G P L M S L I P G L L K - - - - - - - - - - - *	18	1838.35	6	33	89	2	Johnson et al. (2010)
M-PONTX-Dq4a	*Dinoponera quadriceps* (ant)	G L K D W W N K H K D K I V E W K D S G K A G L N A A - - - - - - *	27	3094.48	0	11	48	3	Cologna et al. (2013)
M-ECTX-Eb2b	*Ectatomma brunneum* (ant)	- - - - F W G A L V A G L - - - A P K V A I G I K W A I N K K G	25	2608.18	4	24	80	5	Pluzhnikov et al. (2014)
UyCT3	*Urodacus yaschenkoi* (scorpion)	I L S A I W S G I K S L F - *	13	1433.74	8	31	69	2	Luna-Ramírez et al. (2013)
UyCT5	*Urodacus yaschenkoi* (scorpion)	I W S A I W S G I K G L L - *	13	1442.75	8	31	77	2	Luna-Ramírez et al. (2013)

Figure S10. Alignment of U-MYRTX-MANr1 with peptide sequences found in venoms of other arthropods or defensive skin secretions of frogs, using Clustal Omega [3]. Amino acid sequence relationships with and without conservative substitution are expressed as either sequence similarity (S%) or sequence identity (I%) using the online tool, 'Sequence Identity And Similarity' [4]. Percentages are calculated in relation to the length of the largest peptide sequence. Identical amino acids are highlighted in green and conservative substitutions in blue. Parameters for conservative substitutions: positive (R, K, H), negative (D, E) and hydrophobic (V, I, L, A, F, P, M, W, G). Additional parameters: amino acid sequence length (aa), molecular weight (MW), the share of hydrophobic amino acids (HP%) and net charge (nc). Asterisks indicate C-terminal amidation of the corresponding peptide.

References

1. Senko, M.W.; Beu, S.C.; McLaffertycor, F.W. Determination of monoisotopic masses and ion populations for large biomolecules from resolved isotopic distributions. *J. Am. Soc. Mass Spectrom.* **1995**, *6*, 229–233.
2. Akey, D.H.; Beck, S.D. Continuous Rearing of the Pea Aphid, *Acyrthosiphon pisum*, on a Holidic Diet. *Ann. Entomol. Soc. Am.* **1971**, *64*, 353–356, doi:10.1093/aesa/64.2.353.

3. Sievers, F.; Wilm, A.; Dineen, D.; Gibson, T.J.; Karplus, K.; Li, W.; Lopez, R.; McWilliam, H.; Remmert, M.; Söding, J. Fast, scalable generation of high-quality protein multiple sequence alignments using Clustal Omega. *Mol. Syst. Biol.* **2011**, *7*.
4. Immunomedicine Group. Sequence Identity And Similarity (SIAS). Availabe online: http://bio.med.ucm.es/Tools/sias.html (accessed on 01 July 2019).

© 2019 by the authors. Submitted for possible open access publication under the terms and conditions of the Creative Commons Attribution (CC BY) license (http://creativecommons.org/licenses/by/4.0/).
